# Structural determinants of specificity and regulation of activity in the allosteric loop network of human KLK8/neuropsin

**DOI:** 10.1038/s41598-018-29058-6

**Published:** 2018-07-16

**Authors:** Mekdes Debela, Viktor Magdolen, Wolfgang Skala, Brigitta Elsässer, Eric L. Schneider, Charles S. Craik, Martin L. Biniossek, Oliver Schilling, Wolfram Bode, Hans Brandstetter, Peter Goettig

**Affiliations:** 1 Max-Planck-Institut für Biochemie, Proteinase Research Group, 82152 Martinsried, Germany; 2 Klinische Forschergruppe der Frauenklinik, Klinikum rechts der Isar der TU München, 81675 München, Germany; 3 Division of Structural Biology, Department of Biosciences, University of Salzburg, 5020 Salzburg, Austria; 4 Department of Pharmaceutical Chemistry, University of California, San Francisco, CA 94158 USA; 5 Institute of Molecular Medicine and Cell Research, University of Freiburg, 79104 Freiburg, Germany; 6 BIOSS Centre for Biological Signalling Studies, University of Freiburg, 79104 Freiburg, Germany; 7 German Cancer Consortium (DKTK), German Cancer Research Center (DKFZ), 69120 Heidelberg, Germany; 8 Present Address: Department of Biochemistry, University of Cambridge, Cambridge, CB2 1GA UK

## Abstract

Human KLK8/neuropsin, a kallikrein-related serine peptidase, is mostly expressed in skin and the hippocampus regions of the brain, where it regulates memory formation by synaptic remodeling. Substrate profiles of recombinant KLK8 were analyzed with positional scanning using fluorogenic tetrapeptides and the proteomic PICS approach, which revealed the prime side specificity. Enzyme kinetics with optimized substrates showed stimulation by Ca^2+^ and inhibition by Zn^2+^, which are physiological regulators. Crystal structures of KLK8 with a ligand-free active site and with the inhibitor leupeptin explain the subsite specificity and display Ca^2+^ bound to the 75-loop. The variants D70K and H99A confirmed the antagonistic role of the cation binding sites. Molecular docking and dynamics calculations provided insights in substrate binding and the dual regulation of activity by Ca^2+^ and Zn^2+^, which are important in neuron and skin physiology. Both cations participate in the allosteric surface loop network present in related serine proteases. A comparison of the positional scanning data with substrates from brain suggests an adaptive recognition by KLK8, based on the tertiary structures of its targets. These combined findings provide a comprehensive picture of the molecular mechanisms underlying the enzyme activity of KLK8.

## Introduction

Initially, kallikrein-related peptidase 8 was named neuropsin, since the mouse ortholog *Klk8* or *mK8* had been cloned from mouse brain, in particular as hippocampus cDNA^1^. Similarly, the human *KLK8* gene was cloned from human cDNA^2^, while expression of the KLK8 protein was detected in skin, esophagus, tonsils, salivary gland, breast milk, cervico-vaginal fluid, and in ovarian cancer extracts^3,4^. KLK8, formerly termed hK8, belongs to the 15 human kallikrein-related peptidases of the trypsin-like family S1A, with the MEROPS code S01.244^5^. It is even present in primitive mammals, such as platypus and wallabies^6^. KLK8 is secreted as an inactive pro-enzyme, whereby a signal peptide is cut off, followed by propeptide cleavage at the Lys15-Val16 bond, releasing active enzyme^7^. First hints of a specific function came from mouse *Klk8* expression in the limbic system, with mRNA level changes in pyramidal neurons upon epileptic seizures^8,9^. Similarly, KLK8 mRNA transcripts were found in the human hippocampus and neocortex^10^.

Further studies suggested that mouse Klk8 (mKlk8) participates in important brain processes, such as neural plasticity and memory formation, whereby long term potentiation (LTP) and the formation of synaptic boutons in mice require the active mKlk8 protease, which remodels the extracellular matrix^11^. LTP processes at synapses of higher mammals involve a complicated interplay of KLK8/neuropsin with the proteases tPA, plasmin, MMP-9, ADAM-10, and neurotrypsin^12^. Also, during LTP the activity of voltage-gated calcium channels correlates with elevated extracellular Ca^2+^ concentration, which has a stimulatory effect on the enzymatic activity of KLK8^7,13^. At synapses it cleaves the cell adhesion molecule L1CAM, neuregulin-1, a signal ligand of the ErbB4 receptor, and the tyrosine kinase EphB2, which modulates the expression of the NMDA receptor^12^. Since single nuclear polymorphisms of KLK8 have been discovered in humans with bipolar disorder and learning disability, a similar role in synaptic processes as in mice can be assumed^11^. Real time PCR results demonstrated expression of KLK8 in all parts of the developing embryo brain, while the expression in adult brain is much more restricted^14^. Remarkably, the splice variant KLK8-II from human brain, with a 45 residue longer signal peptide^14^, was discovered in archaic hominins, such as Neanderthals and Denisovans, but is absent chimpanzees^15^. Thus, it has been hypothesized that the splice variant KLK8-II is a distinguishing feature of humans compared with the great apes and other primates^16^.

Apart from its functions in brain, KLK8 contributes to wound healing, as demonstrated with knockout mice^17,18^. Also, elevated KLK8 levels were found in the inflamed psoriatic plaques of humans and in a corresponding mouse psoriasis model^19,20^. KLK8 and its mouse ortholog degrade extracellular matrix proteins, such as fibronectin and type-IV collagen^21,22^. Several natural inhibitors of KLK8 have been described, such as SerpinB6, SerpinB9 and the protein C inhibitor, as well as the skin-specific Kazal-type serine protease inhibitor SPINK9 blocks human KLK8 activity^23^.

In order to explore human KLK8, the recombinant enzyme from an *E*. *coli* expression system was used for X-ray and enzymatic studies, which are the basis for a comparison with the structure of mKlk8^24^ and for a mechanistic analysis of the structure-functional modules of this intriguing protease.

## Results

### Extended substrate specificity profiles of KLK8

**Figure 1:**
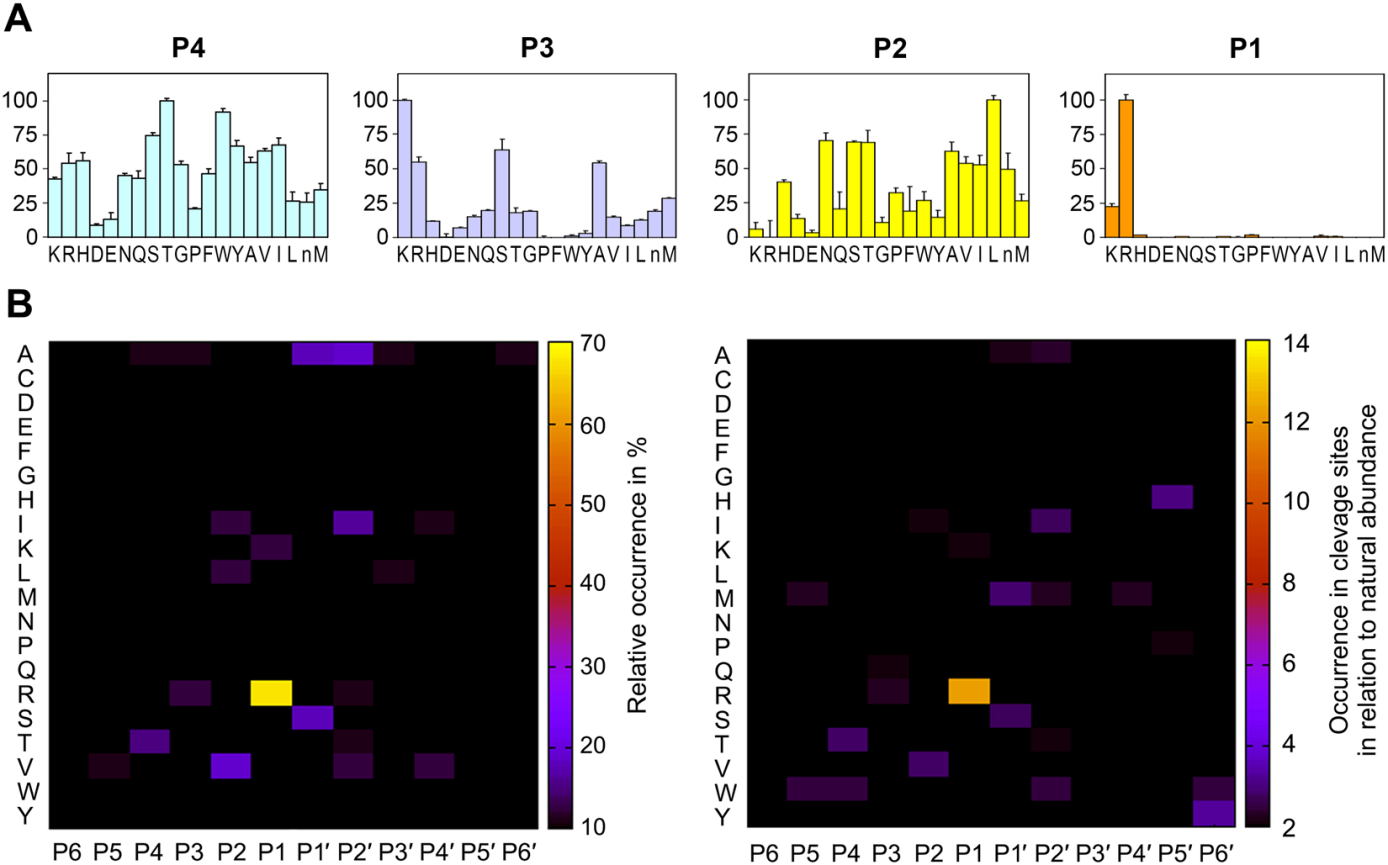
Specificity profiling with a positional scanning and proteomic identification of cleavage sites. (**A**) Peptide substrate specificity of KLK8 measured by positional scanning for peptide positions P4 to P1. The height of the columns represents substrate cleavage rates by released ACC, while the x-axis indicates the amino acids in one-letter code, whereby n represents norleucine, which substitutes Cys. (**B**) PICS heat maps with amino acids listed in alphabetical order along the y-axis and peptide positions run from P6 to P6′ on the x-axis. The left panel displays cleavages in 73 substrates and the right panel contains corrected values for the natural abundance of each residue. Both PICS and PSSCL agree in a moderate preference for Thr and Trp in P4 and for basic residues in P3, such as Lys or Arg. Aliphatic residues Leu, Val, and Ile are found by both methods in P2, whereby Val dominates in the corrected PICS, while PSSCL ranks the polar Asn, Ser, and Thr relatively high. The major primary specificity for Arg over Lys in P1 is found by both methods, albeit stronger in PICS. The corrected PICS data show a preference for Ser and Met in P1′, for Ile and Trp in P2′, and no distinct specificity for P3′ and P4′. By contrast, S5′ and S6′ subsites are moderately specific for His and Tyr.

Active, non-glycosylated KLK8 was prepared from a chimeric construct expressed in *E*. *coli*, refolded and activated by enterokinase^25^. According to SDS-PAGE analysis, this recombinant KLK8 was more than 95% pure, and its integrity was shown by N-terminal sequencing and mass spectrometry. Since only scattered information on natural or synthetic KLK8 substrates was available, the extended substrate specificity of KLK8 was determined by a positional-scanning screening of a synthetic diverse tetrapeptide library (PSSCL) and the proteomic identification of cleavage sites (PICS), which employs a peptide library from digested *E*. *coli* proteins^26,27^. Regarding the non-prime side, KLK8 possesses a typical tryptic S1 subsite and cleaves mainly at P1-Arg residues and with less than 25% the efficiency at Lys in both approaches (Fig. 1A,B). Both methods indicate that the S2 pocket prefers the aliphatic residues Leu, Val, and Ile, while PSSCL ranks polar residues, such as Asn, Ser, and Thr relatively high. Unusual for trypsin-like serine proteases the S3 subsite favors the basic side chains Lys and Arg, besides Ser and Ala in case of PSSCL and only Arg according to PICS. PSSCL shows that the S3 subsite disfavors Asp and Glu as P3 residues, while the overall less specific S4 subsite accommodates best Thr and Trp significantly above average. The PICS method alone provided information on the prime side specificity, where the S1′ subsite prefers the small or polar residues Ser and Ala, the S2′ pocket exhibits a preference for Ala, along with the aliphatic residues Ile and Val, which also appears to be better accepted in the S4′ subsite than other residues. A moderate preference for P5′-His and P6′-Tyr shows up after correction for the natural occurrence of amino acids in *E*. *coli* proteins (Fig. 1B). Based on the PSSCL results for the range of P1 to P4 residues, optimized fluorogenic 7-amino-4-methyl coumarin (AMC) and 7-amino-4-carbomoylmethyl coumarin (ACC) substrates were synthesized.

### Ca^2+^ stimulation and Zn^2+^ inhibition of recombinant KLK8 activity

Active-site titration with the burst reagent p-nitrophenyl-p’-guanidino-benzoate (NPGB) revealed that 62% of the recombinant KLK8 was active. This value was used as molar fraction of active protease for the calculation of the turnover number (k_cat_). The ideal substrates Ac-Thr-Lys-Leu-Arg-AMC and Ac-Thr-Lys-Leu-Arg-ACC were checked for their turnover by KLK8 and compared with other tetrapeptidyl substrates, regarding their enzyme kinetic parameters k_cat_, K_M_, and the resulting catalytic efficiency (Table 1). For example, Ac-Thr-Lys-Leu-Arg-ACC exhibited a Michaelis constant (K_M_) around 15 µM, a k_cat_ around 45 s^−1^ and a catalytic efficiency k_cat_/K_M_ = 3 137 000 M^−1^ s^−1^, whereas the previously described best KLK8 substrate Bz-Val-Pro-Arg-AMC had a k_cat_/K_M_ = 20 280 M^−1^ s^−1^ ^7^. Both optimized substrates turned out to be at least 10-times more active than commercial substrates such as Ac-Thr-Lys-Pro-Arg-AMC, whereas Tos-Arg-AMC was not cleaved at all. No turnover was seen for Boc-Glu-Lys-Lys-AMC, which is cleaved by mKlk8 to some extent^28^, or for Ac-Trp-Glu-Asp-Arg, in line with the PSSCL findings that basically exclude negatively charged P2 and P3 residues.

**Table 1:** Enzyme kinetic parameters of KLK8 with various synthetic substrates.

	k_cat_ (s^−1^)	K_M_ (μM)	k_cat_/K_M_ (M^−1^s^−1^)
Ac-Thr-Lys-Leu-Arg-ACC	48.0 ± 2.1	15.3 ± 1.5	3 137 300 ± 333 800
Ac-Thr-Lys-Leu-Arg-AMC	43.9 ± 6.2	14.4 ± 0.2	3 049 000 ± 432 600
Z-Val-Val-Arg-AMC	6.5 ± 0.9	57.5 ± 10.7	113 000 ± 26 200
Boc-Val-Pro-Arg-AMC	8.8 ± 0.4	434.0 ± 100.2	20 300 ± 4 800
Bz-Pro-Phe-Arg-pNA	2.0 ± 0.5	430.0 ± 68.0	4 650 ± 1 380

Zinc ions have a strong inhibitory effect on the hydrolytic activity of KLK8. At pH 8.0 and 20 °C, we determined an IC_50_ value of 3.6 ± 0.3 µM (see section on the zinc binding 99-loop), close to the IC_50_ value of 3.3 µM measured for different KLK8 preparation at pH 8.5 and 37 °C^7^. Eadie-Hofstee plots of the activity and substrate concentration at different Zn^2+^ concentrations indicate that the mechanism of inhibition is non-competitive, which suggests that Zn^2+^ binds the free and substrate saturated enzyme with similar affinity.

In contrast to Zn^2+^, Ca^2+^causes an increase of the hydrolytic activity of KLK8 with the para-nitroanilide Bz-Pro-Phe-Arg-pNA exhibiting a maximum around 1 mM which agrees with a previous publication^7^. However, KLK8 cleaved the optimal substrates Ac-Thr-Lys-Leu-Arg-ACC and Ac-Thr-Lys-Leu-Arg-AMC with a 2-fold increase of activity at 200 to 300 µM Ca^2+^. For the substrate Bz-Pro-Phe-Arg about 60% higher KLK8 activity was observed in the presence of 1 mM Ca^2+^ at 37 °C, while KLK8 that had been preincubated with 5 µM Zn^2+^ showed nearly 70% increase of activity upon addition of Ca^2+^.

### Overall structure of KLK8

Originally, one form of KLK8 could be crystallized in the presence of the aldehyde inhibitor leupeptin. Several data sets were collected of these crystals in space group P2_1_2_1_2_1_, with one molecule per asymmetric unit and a maximum resolution of 2.3 Å. The corresponding KLK8 structure will be referred to as KLK8-Ca. Upon soaking the crystals with ZnCl_2_, one cell constant approximately doubled in length, accompanied by a transition to space group P2_1_, with four molecules per asymmetric unit. The corresponding 2.1 Å structure of KLK8, in which Zn2+ was not clearly identified, is referred to as KLK8-leup and resembles largely the KLK8-Ca molecule.

**Figure 2:**
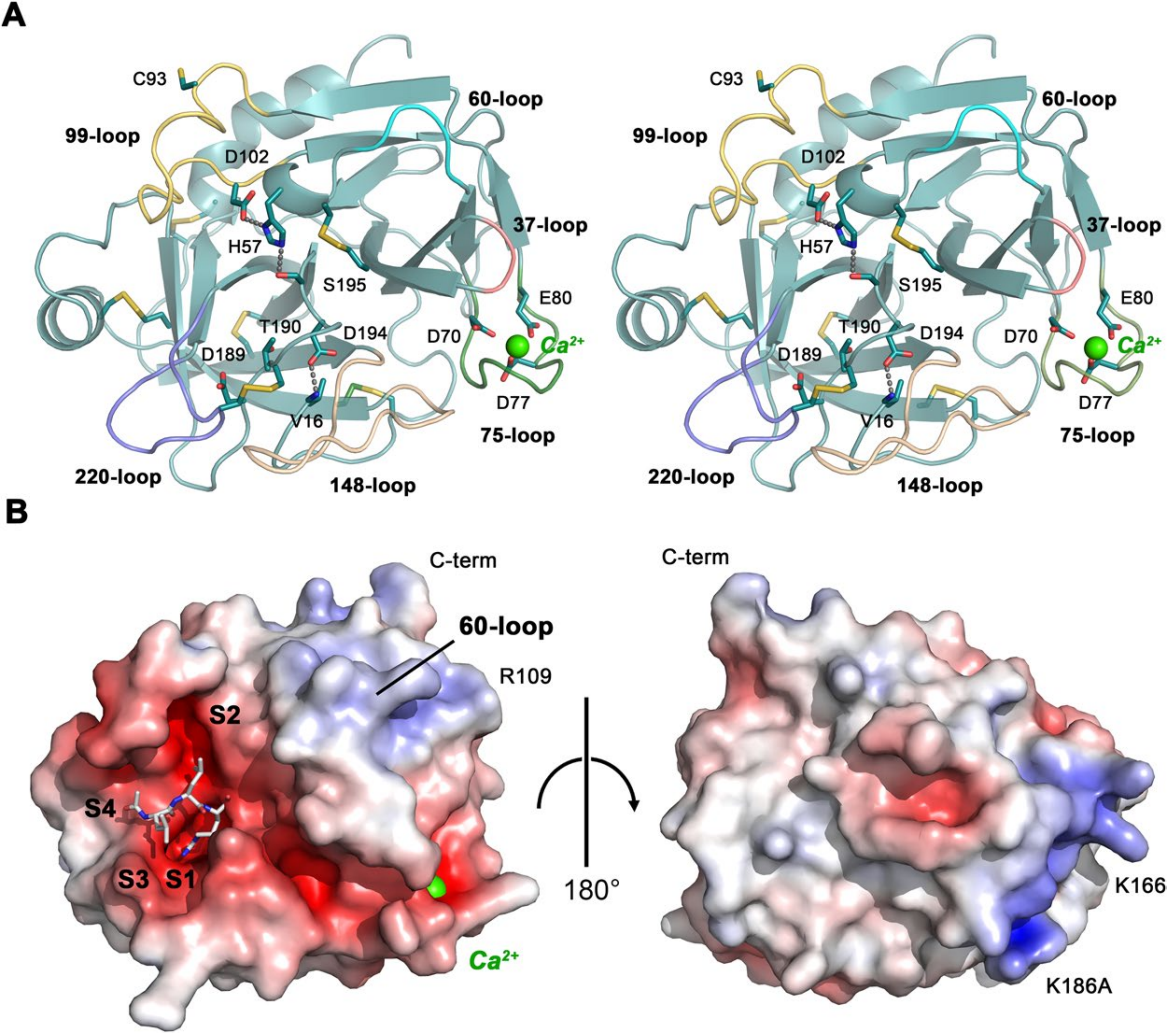
Overall structure of KLK8-Ca. (**A**) Ribbon stereo plot of KLK8-Ca shown in standard orientation. The side chains of the catalytic triad, the activating salt bridge Val16-Asp194, the major specificity-determining Asp189 and Thr190, as well as the six disulfide bridges and Cys93 are shown as sticks (carbon teal, nitrogen blue, oxygen red, and sulfur yellow). Also, the three major ligands of Ca^2+^ (green sphere) are depicted, namely Asp70, Asp77 and Glu80. Functionally important loops are displayed in different colors with labels. (**B**) Surface model of KLK8 with inhibitor and Ca^2+^. The left panel shows KLK8 in standard orientation with a stick model of leupeptin, occupying the subsites S4 to S1. The electrostatic surface potential is depicted red for negative and blue for positive potential in the range −10 e/k_B_T to +10 e/k_B_T. The right panel shows the KLK8 backside, rotated by 180°. The 60-loop and the area around Arg109 correspond to the thrombin anion binding exosite I, while the positive patch at the back around Lys166 and Lys186A has no counterpart.

As in most trypsin-like serine proteinases the KLK8 chain consists of two six-stranded β-barrels that can be considered as half-domains (Fig. 2A). At their interface the catalytic triad of Ser195, His57 and Asp102 is situated above the specificity subsites S4 to S4′, aligned from left to right in the standard orientation. Compared to the coordinates of mKlk8 (PDB code: 1NPM) human KLK8 exhibits a relatively low root mean square deviation (RMSD) of 0.63 Å for 222 equivalent residues atoms, agreeing well with respect to 73% identical residues^7^. Similar to other mature KLKs, the N-terminal α-ammonium group of Val16 forms the crucial salt bridge to the side chain carboxylate of Asp194, which stabilizes the oxyanion hole and a properly shaped S1 pocket (Fig. 2A). Also, KLK8 contains the KLK-specific cis-Pro219 and six disulfide bridges.

The active site cleft from the non-prime specificity pockets S4 to S1 and S1′ to S4′ is predominantly negatively charged (Fig. 2B). Several basic residues cluster around the 60-loop in a positively charged surface patch, extending above the prime side region of the active-site cleft in standard orientation. Together with the positively charged patch around Arg109 this area resembles the more extended basic regions of KLK5 and KLK7 and the functionally important anion binding exosite I of thrombin^29^.

### Active-site cleft and specificity pockets

**Figure 3:**
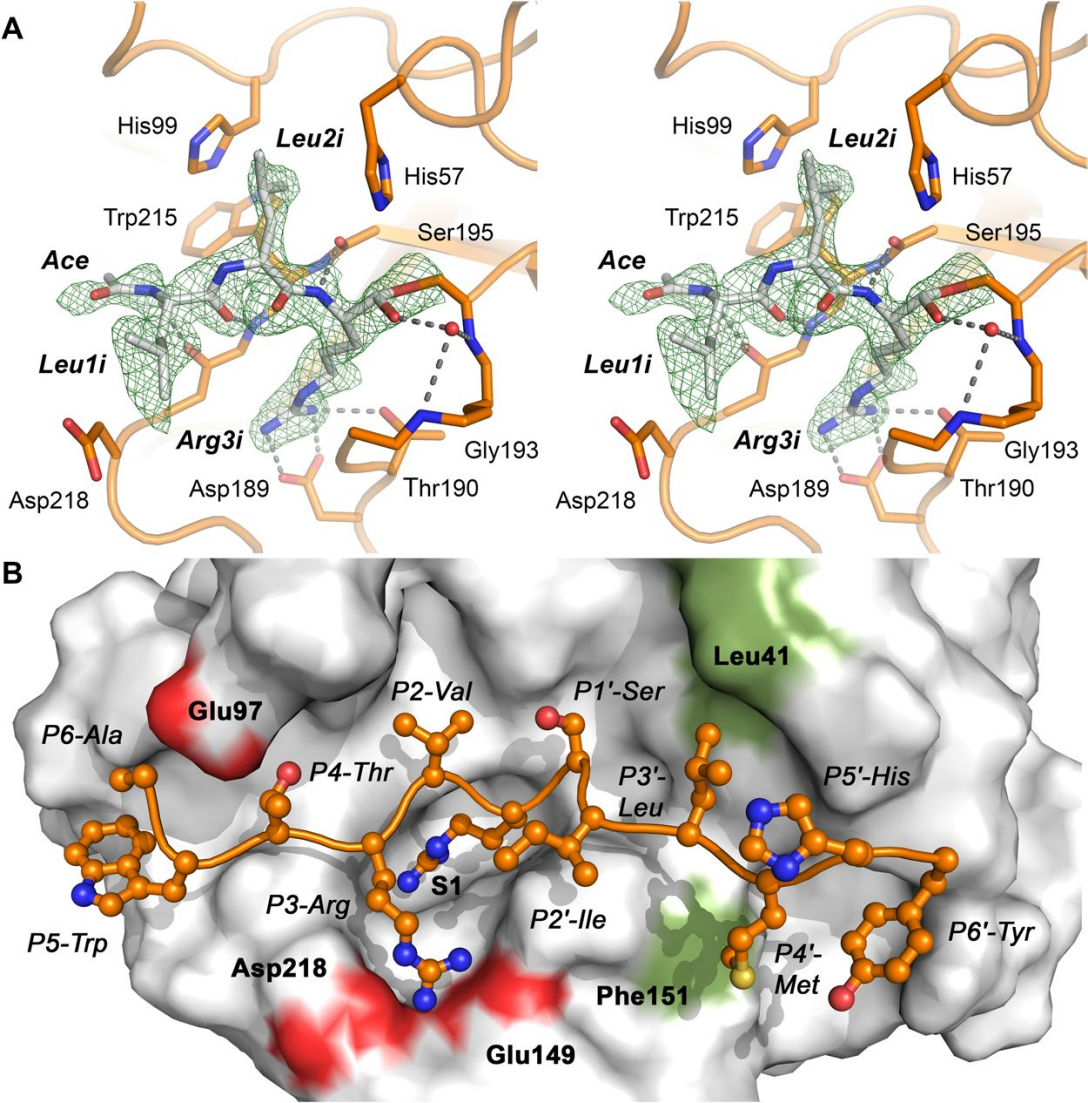
Leupeptin inhibitor and ideal substrate binding to the active site. (**A**) Stereo model of leupeptin in KLK8-leup, with the most relevant interacting residues displayed as sticks, e.g. the covalently linked Ser195. The electron density is a 2Fo-Fc map shown as green grid, contoured at 1.0 σ, hydrogen bonds are represented by grey dots. A water molecule (red sphere) occupies the oxyanion hole at the NH groups of Gly193 and Ser195 in this chain. (**B**) Molecular docking of an ideal substrate to KLK8 according to PICS profiling for residues P6 to P6′. Binding of residues P4 to P4′ agrees well with the specificity profiles. Moreover, the calculated interactions of P4-Thr with Glu97 and P3-Arg with Glu149 and Asp218 (red patches) can explain the preference of the S4 subsite for Thr over Trp, and the unique S3 preference for basic side chains. Binding of the hydrophobic residues P3′-Leu and P4′-Met may depend on the subsite shaping residues Leu41 and Phe151 (green patches).

Both KLK8 structures exhibit a similar active site architecture, whereby in the KLK8-Ca structure the substrate binding cleft is occupied by several water molecules, located mostly at polar or charged positions of the specificity pockets. By contrast, the P2_1_ form of KLK8-leup contains four copies of the aldehyde inhibitor leupeptin, with the sequence acetyl-Leu1i-Leu2i-Argininal3i. For this reversible inhibitor an IC_50_ of 66 µM was reported for KLK8 from insect cell expression, while it seems less than 10 µM for mKlk8^7,28^. Leupeptin binds covalently to Ser195 as analogue of an acyl intermediate in the canonical conformation (Fig. 3A). The P1-Arg3i aldehyde group in KLK8-leup is covalently linked to Ser195 Oγ as hemiacetal, with the oxygen occupying the oxyanion hole in the four KLK8 copies. This orientation of the hemiacetal oxygen corresponds to one of the two alternative conformations observed in a trypsin leupeptin complex (PDB code 1JRT)^30^. It forms hydrogen bonds to the backbone amide N-H atoms of Gly193 and Ser195 or a nearby water at average distances of 3.3 Å, while the other conformation with a hydrogen bond to the Nε2 atom of His57 is not observed.

Similar to a substrate, leupeptin forms a short antiparallel β-sheet with two backbone hydrogen bonds between P3-Leu1i and Gly216, and the amide of P1-Arg3i and the carbonyl oxygen of Ser214 (Fig. 3A). The Arg3i side chain is buried in the S1 specificity pocket, which is rather hydrophobic in the upper region, whereas at the bottom the side-chains of Thr190, Ser217, Tyr228, and a water molecule bound to the Ser217 carbonyl O contribute to a polar environment. The negatively charged carboxylate of Asp189 forms a tight salt bridge to the positively charged Arg3i guanidyl group. Both positional scanning and PICS analysis corroborated that Arg is strongly favored over Lys as P1 residue (Fig. 1). This finding can be explained by the presence of Thr190, which enhances the specificity for P1-Arg similar to Ser190 of many trypsin-like proteinases, whereas an Ala190 would shift the P1 preference towards Lys^31^.

The funnel-shaped S2 subsite of KLK8 is bordered by the hydrophobic side-chains of His57, His99, and partially Trp215, which explains very well the accommodation of the Leu2i side-chain of the inhibitor and the preference for the aliphatic Val and Leu residues in the substrate profiling (Fig. 1). Also, the hydroxyl group of Tyr94 may interact with polar residues, such as Asn or Ser, which are accepted according to the PSSCL. Overall, this subsite appears to be quite flexible and adopts in all five KLK8 molecules different side chain conformations, in particular for His99, while water molecules sit at varying positions.

Although most P3 side chains of serine protease substrates extend to the bulk solvent, two major alternative conformations are possible for Leu1i and, consequently, for the acetyl group. In three KLK8-leup copies, the Leu side chain prefers the canonical conformation in the bulk solvent, whereby its backbone NH can form a hydrogen bond to the carbonyl O of Gly216 as in KLK5^32^, whereas in one copy it occupies the S4 pocket as in trypsin^30^. As Leu is not a preferred P3 residue according to PSSCL and PICS, it is difficult to relate structural and functional information for the unusual specificity of the S3 subsite for basic residues (Fig. 1). The variable arrangement of the predominantly hydrophobic His99, Trp215, Tyr172 and the more polar Gln175 and Glu97 may suffice to explain the relatively low specificity for P4 residues, including the uncommon preference for Thr and Trp, and the rejection of negatively charged P4 residues.

A molecular docking calculation for an ideal peptide substrate of KLK8, derived from the PICS data, resulted in proper binding of P4 to P4′ (Fig. 3B). Besides the expected accommodation in the S2 to S1 pockets, the interaction of the Arg guanidyl group by the carboxylate groups of Asp218 and Glu149 explains the specificity profiles for the preferred basic P3 residues. Also, a polar interaction of the P4-Thr with Glu97 may be the basis for the unusual S4 specificity. The S1′ subsite is a shallow pocket that is mostly shaped by two main chain stretches with polar character, while the hydrophobic bottom consists of the disulfide Cys42-Cys58, which agrees with a moderate preference for P1′-Ser and Met. Eventually, the accommodation of the aliphatic P2′-Ile, P3′-Leu and P4′-Met depends on the hydrophobic environment, such as Leu40, Leu41, Lys60 and Phe151 bordering the S2′ to S4′ subsites, respectively. The adjacent S5′ and S6′ subsites possess a mixed character, which tolerates aromatic, but also charged or polar residues, such as His and Tyr, while still putative prime side subsites may extend even beyond the 75-loop.

### The Ca^2+^ binding 75-loop

**Figure 4:**
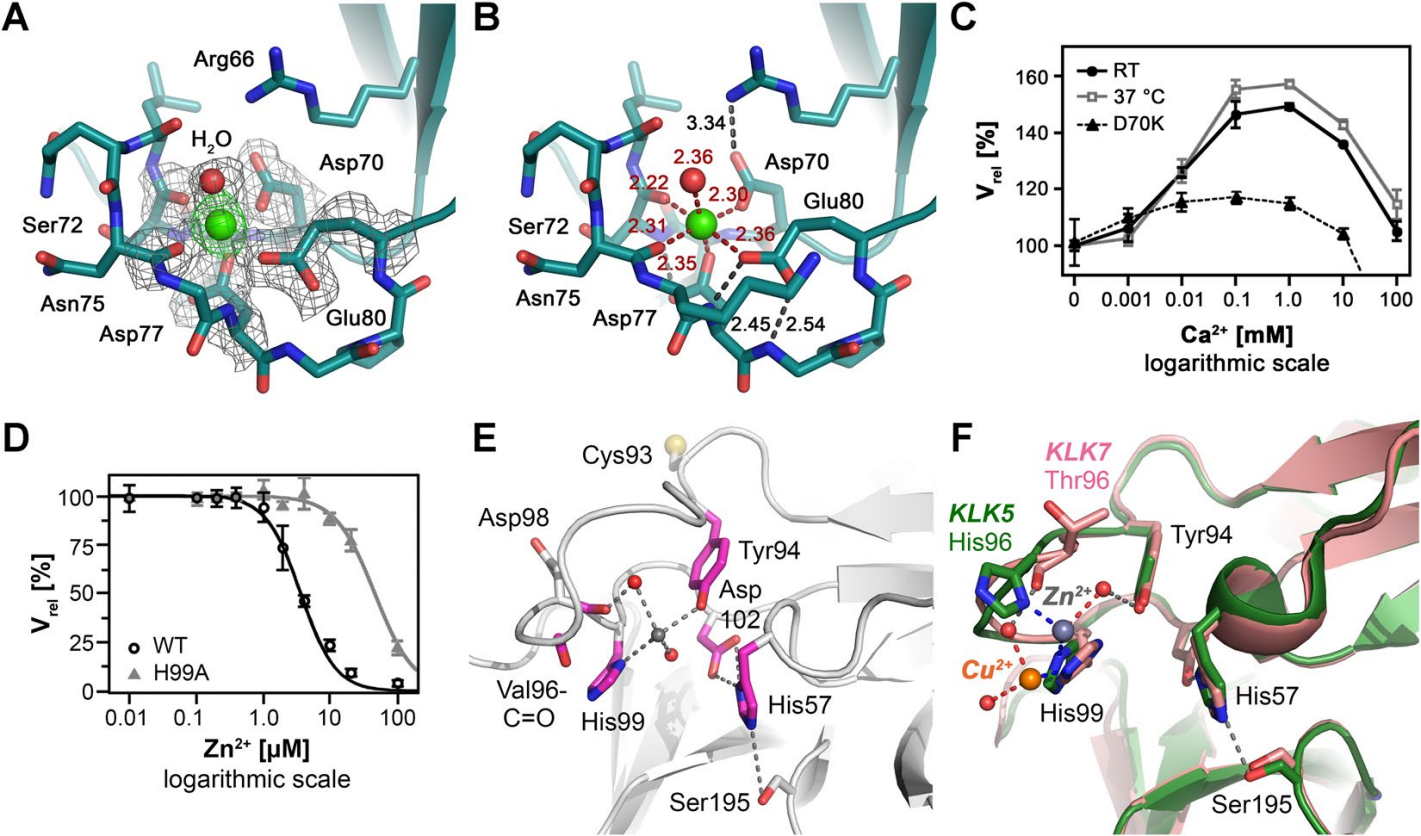
The Ca^2+^ and Zn^2+^ binding loops of KLK8 as regulatory modules. **(A**) The 75-loop of KLK8-Ca with Ca^2+^ ligands surrounded by the 2F_o_-F_c_ electron density map, contoured at 1.0 σ. Ca^2+^ is depicted in green with electron density contoured at 3.0 σ, while the water ligand is shown as red sphere. (**B**) Ligand bonds to Ca^2+^ are shown as red dots and relevant hydrogen bonds as black dots, including distances in Å. The excess charge of three carboxylates is compensated by Arg66 near Asp70 and hydrogen bonds of Asp77 and Glu80 with backbone N-H groups. (**C**) The Ca^2+^ stimulation of the KLK8 activity is maximal in the range 100 µM to about 1 mM. In the D70K variant no significant stimulatory effect of Ca^2+^ was observed. (**D**) The mutant His99Ala confirmed the inhibitory Zn^2+^ site with a 13-fold higher IC_50_, i.e. 47 µM versus 3.6 µM of wild-type KLK8. (**E**) In the 99-loop, Cys93 can be excluded as potential Zn^2+^ ligand, since Zn^2+^ inhibition of the Cys93Ser variant was unchanged compared to the wild-type. A water molecule (grey) between His99 and Tyr94 in KLK8-leup is in a suitable position to bind Zn^2+^. The variant Tyr94Phe has a 2-fold higher IC_50_, suggesting a minor role as Zn^2+^ ligand for Tyr94. Hydrogen bonds are shown as grey dots. (**F**) Two complex structures of KLK5 and KLK7 with Zn^2+^ and Cu^2+^ feature ligands for cations, such as the carbonyl O of residue 96 and Tyr94. The KLK7-His99Ala mutant nearly abolished the non-competitive Zn^2+^ inhibition, as for KLK8. This inhibition type can be explained by Zn^2+^ binding to the His57 side chain, which has to switch from the catalytic triad. A rotation of His99 would bring both ligands in ideal distances to Zn^2+^ ^36^.

Among the loops shaping the active-site cleft of KLK8, the 75- or “calcium binding”-loop is the most distant from the catalytic triad. This loop is named after the central residue, but it is also known as the 70–80-loop. The 75-loop of KLK8-Ca exhibits an ideal coordination geometry compared to the slightly deviating KLK8-leup loops and holds the Ca^2+^ ion with six ligands at an average distance of 2.3 Å, which is slightly below standard values (Fig. 4A,B)^33^. Among them are the side chain carboxylate groups of Asp70, Asp77, and Glu80, which provide a negative charge excess with respect to the divalent cation. Since the Oδ1 and Oδ2 of Asp77 bind the backbone N-H group of Ser72 at a distance of roughly 3.1 Å, some portion of the third negative charge is compensated. Similarly, the carboxylate of Glu80 binds the N-H groups of Asp77 and Gly78 with its Oε2 and Oε1 atoms, respectively. Also, the positively charged side chain of Arg66, appears to compensate some portion of the negative excess charge by a contact of the Nη1 to the Oδ2 of Asp70 at around 3.3 Å, albeit not with an ideal geometry. Additionally, the Ca^2+^ ion is bound with an average distance of 2.3 Å by the carbonyl O atoms of Ser72 and Asn75, as well as by one water molecule. The Ca^2+^ coordination sphere is octahedral, as it is known for coagulation factors or trypsin where the 75-loop binds Ca^2+^ via three glutamates, including a mediating water^34^. By introducing the substitution Asp70Lys in the 75-loop, the stimulatory effect of Ca^2+^ was essentially abolished in enzymatic assays with Boc-Val-Pro-Arg-AMC or Bz-Pro-Phe-Arg-pNA compared with normal activity (Fig. 4C). The function of Ca^2+^ bound to the 75-loop can be dual as in trypsin, where it serves both to stabilize the enzyme and enhance the activity^35^.

### Zn^2+^ binding in the 99-loop

The 99-loop of KLK8 exhibits an intermediate length between those of KLKs 4, 5, 6 and 7 and the long, exposed “kallikrein-loops” of KLKs 1 to 3 with an insertion of 11 residues^23^. Guided by the crystal structures of murine neuropsin, KLK5 and other KLKs with confirmed Zn^2+^ inhibition we generated mutants in order to investigate the functional role of critical residues located in the 75- and 99-loops^23,24^. The substitution of His99Ala resulted in an active KLK8 variant that exhibited a 13-fold higher IC_50_ (46.7 ± 3.2 µM) for Zn^2+^ inhibition with respect to the wild type (Fig. 4D). Lack of the hydroxyl group in the variant Tyr94Phe resulted in a doubled IC_50_ (6.9 µM), which hints to a role as secondary or water bridged ligand of Zn^2+^ (Fig. 4E). Since cysteines are favored Zn^2+^ ligands, the residual inhibition of the His99Ala mutant could depend on Cys93 (Fig. 4E), which was mutated to a serine. However, the Cys93Ser variant was as active as the wild type and displayed similar inhibition by Zn^2+^, which excludes Cys93 as ligand, since Ser is as Tyr not an ideal ligand for Zn^2+^ ^36^. This finding suggests that substrates still can bind to the active site in the inhibited state, while the inactivation is based on another process, such as a the disruption of the catalytic triad, which was found in the crystal structure of rat tonin (klk2) in complex with Zn^2+^ (PDB code 1TON)^37^. Similarly, His57 in rat trypsin relocated from the triad to bind Cu^2+^ together with an engineered His96 (1AND)^38^. Since both KLK5 and KLK7 structures exhibit either Zn^2+^ or Cu^2+^ bound to the equivalent His99 ligands and His96 or the Thr96 carbonyl O, other possible ligands are His57 or perhaps Asp102 (Fig. 4F). At least, we can narrow down the most likely Zn^2+^ binding residues to the region around His99, Tyr94 and His57 in the S2 subsite (Fig. 4F). There, the Tyr94 OH and the carbonyl O atoms of Val96 and Asp98 could serve as additional ligands or secondary ones by bridging water molecules in the coordination sphere of the metal ion.

## Discussion

**Figure 5:**
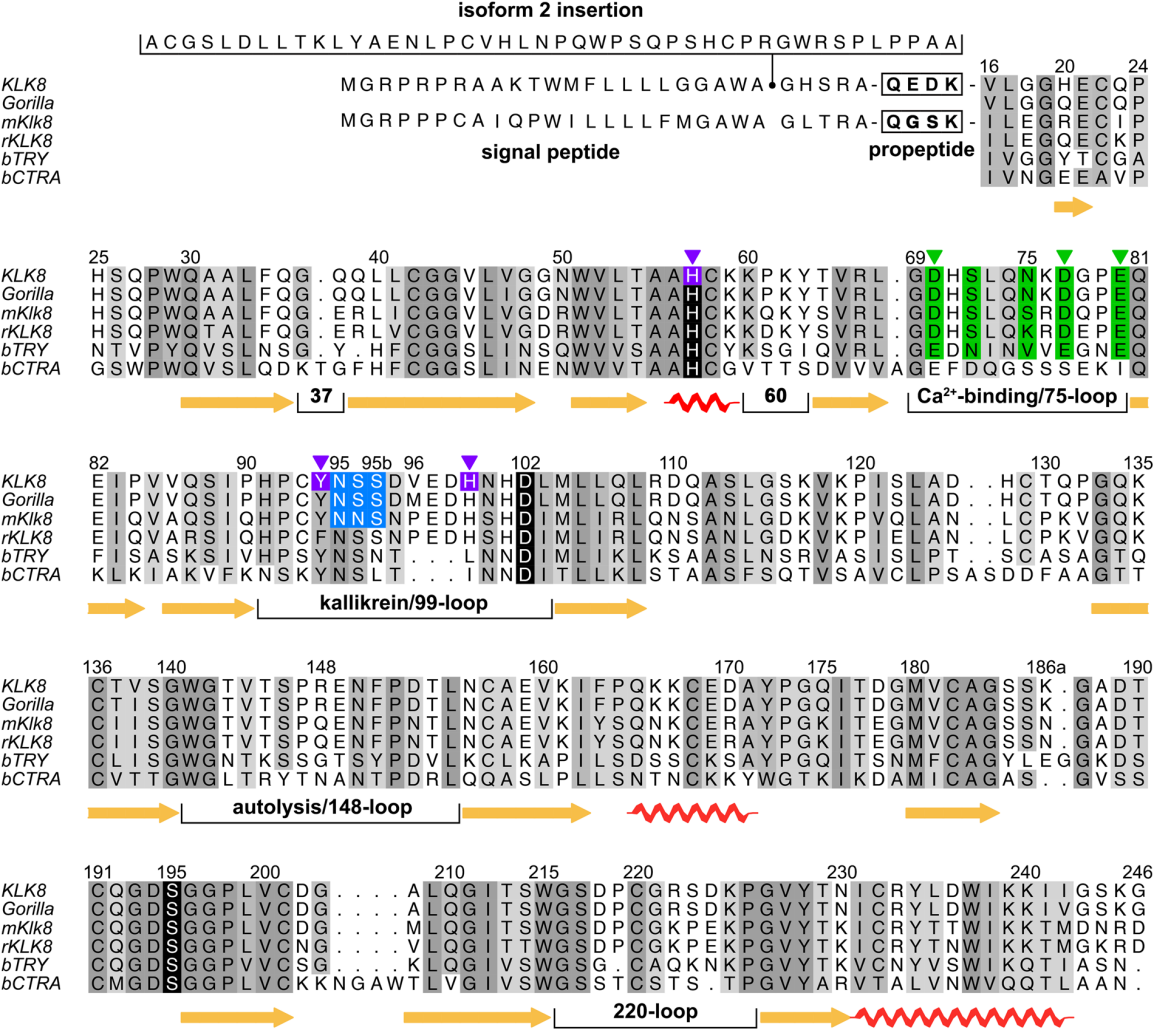
Structure-based sequence alignment of KLK8. Other species are gorilla (*Gorilla gorilla*), mouse (*Mus musculus*) and rat (*Rattus norvegicus*) (mKlk8 and rKLK8), including bovine trypsin (bTRY) and chymotrypsinogen A (bCTRA) as numbering standard. Signal peptides of isoforms precede the propeptide, whereby isoform 2 with a 45 residue insertion is supposed to be unique for humans. Human and chimpanzee KLK8 are identical, whereas the gorilla has three exchanges. Mice share 73% identical residues in KLK8 with humans, similar to rats with about 71%. β-sheets, α-helices, and the short 3_10_-helix (residues 56–59) of KLK8 are indicated by arrows and screws, respectively. Relevant surface loops are labeled according to the central residue. Darker shades of grey represent higher conservation, while the catalytic triad residues are depicted with black background. Ca^2+^-ligands are displayed with green background and Zn^2+^ ligands are shown with magenta background, triangles indicate interacting side chains. The sequons of the glycosylation site at Asn95 are highlighted in blue.

KLK8/neuropsin is among the major suspects that make a substantial difference in human neuronal processes with respect to the corresponding activity in other primate brains. A structure based sequence alignment demonstrates that only three insignificant amino acid exchanges are present in the active KLK8 protease of our second closest relative in evolution, the gorilla, whereas chimpanzees have exactly the same sequence as humans (Fig. 5). Apparently, the major difference in all non-human primates is the lack of KLK8 isoform 2, with a 45 residue longer signal/prepeptide, which could be responsible for altered protein trafficking, as observed for long signal peptides of other protein isoforms^14,39^. In case the insertion is part of an elongated propeptide of pro-KLK8, it might not influence the distorted loop network at all, whereas KLK8 activation might be altered, favoring a more efficient auto-activation as observed for KLKs 2 and 5^40^. Since isoform 2 is mostly expressed in the hippocampus, the special role of KLK8 in learning and memory formation, may be highly important for human consciousness.

Regarding natural KLK8 substrates, pro-KLKs 1, 2, 3, 5, 6, 9, 11, and 12 appear to be physiologically relevant in KLK activation cascades^40^. The growth hormone somatotropin is another interesting natural substrate, as well as single-chain t-PA, which after activation by KLK8 activates plasmin, facilitating cancer cell invasion^22,41^. Among neuronal mouse substrates is the signal protein neuregulin 1 (NRG1), which is cleaved by Klk8 at three sites with P1-Arg^42^. Although KLK8 expression correlates with cleavage of the neural cell adhesion molecule L1-CAM, the specific sites have not been determined yet^43^. However, murine Klk8 cleaves the receptor tyrosine kinase ephrin type-B receptor 2 **(**EphB2) after Arg518, resulting in NMDA receptor dependent synaptic plasticity events in stress response^44^. Recently, KLK8 inhibition was proposed as therapeutic target in Alzheimer’s disease, which is accompanied by elevated KLK8 protein levels, while in a murine Alzheimer’s model Klk8 inhibition by antibodies significantly attenuated the pathology^45^.

Both the synthetic library and the proteomic specificity profiling agree very well regarding the non-prime side, while there is basically no discrepancy with the MEROPS specificity matrix (Fig. 1). Also, the specific interactions of the P4 to P1 residues can be well explained on a structural level by the complex of KLK8 with the leupeptin inhibitor. In the prime side region, the MEROPS matrix is biased by the IVGG motif that is often present at the N-terminus of serine proteases, which are substrates in KLK activation cascades^40^. Otherwise, this part of the matrix, summarized as Ia/vi/gn/G with small letters for lower preference, resembles more the corresponding raw profile of PICS (as/avi/-/v) than the corrected one (ms/iw/-m) (Fig. 1B). Comprising 25 cleavages, the MEROPS matrix is just below the theoretical limit of 30 substrates for a reliable specificity determination within a 95% confidence interval, using the information entropy as measure of specificity^46^. By contrast, the PICS measurement comprised 73 substrate cleavages, which increases the reliability substantially, as evidenced by the concordance with the virtually unbiased positional scanning library results (Fig. 1A).

**Figure 6:**
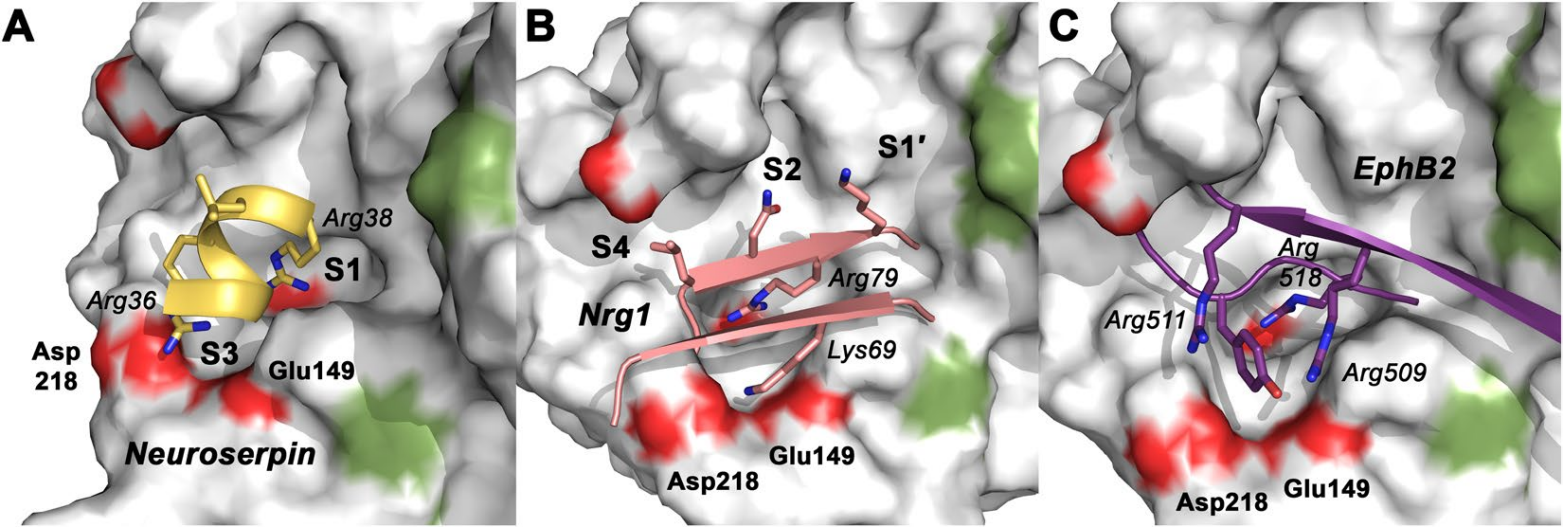
Models of natural protein substrates bound to the active site of KLK8. The molecular surface of KLK8 is shown with red patches of acidic residues and green for hydrophobic ones. (**A**) Human neuroserpin, a serine protease inhibitor from the hippocampus, is cleaved at Arg38 as P1 residue, which is located in the last turn of an α-helix, as observed in the crystal structure (PDB 3FGQ). Apparently, Arg36, Leu37 and Ala39 are suitable P3, P2 and P1′ residues in accordance with our profiling results. Proper binding and turnover by KLK8 requires a transition of the helical turn into a more linear, extended conformation. (**B**) The synaptic regulator neuregulin 1 (NRG1) possesses only P1-Arg79 and P2-Asn78 as KLK8 specific substrate residues at its cleavage site. According to a structure-based homology model, created by SWISS-MODEL^79^ they belong to a β-strand, while the Leu77 side-chain might occupy the S4 instead of the S3 subsite. However, an adjacent β-strand contains Lys69, which is located close to Glu149 and Asp218, substitute the preferred basic P3 side-chain. (**C**) Similarly, a model from SWISS-MODEL of the neuronal KLK8 substrate ephrin type-B receptor 2 (EphB2), exhibits an arrangement of two strands, with P1-Arg518 as only specific residue for cleavage by KLK8. Interestingly, Arg509 and Arg511 might be ideally positioned for electrostatic interaction with Glu149 and Asp218.

A structure based comparison of the specificity matrix and the profiling results with physiological substrates from murine and human neuronal tissue, i.e. NRG1, EphB2, and neuroserpin (SERPINI1), reveals that several cleavage sites are located in disordered regions as it is usually expected, but some comprise secondary structural elements as it was established for other proteases^47^. Some cleavage sites resemble the extended conformation found in surface loops and strands, which also coincide mainly with P1 to P3 sequence that is determined by mainly electrostatic and to some extent by hydrophobic and polar interactions. The cleavage sites KKE^18^R-GSGK of NRG1 and NRL^38^R-ATGE of neuroserpin belong to this category, probably requiring the α-helix of the non-prime side (PDB code 3FGQ) to unwind to some extent (Fig. 6A)^42,48^. Also, the reactive center loop of neuroserpin, which has been suggested as physiological inhibitor of KLK8 in the brain, with the sequence ISM^362^R-MAVL and an α-helical turn could fit in here^49^. However, the cleavage sites ELN^79^R-KNKP of NRG1 and GYG^518^R-YSGK of EphB2 hardly agree with the established substrate specificity of KLK8, except for the presence of P1-Arg^42,44^. Intriguingly, the three-dimensional models of both sites exhibit accessible surface stretches with basic residues in neighbor strands, such as Arg67 and Lys69 in NRG1 or Arg509 and Arg511 in EphB2 (Figs 6B,C). They are in reach of Asp218 and Glu149, which appear to be the determinants of the specificity for basic P3 residues. Thus, only by considering the three-dimensional structure of substrates with large deviations from the ideal recognition sequence, we can explain the preference of KLK8 for its known physiological targets. Since the phenomenon of significantly differing synthetic and natural substrate preferences is well known for other proteases, systematic analyses of similar cases may indeed reveal this type of alternative presentation of preferred cleavage sites as a more general pattern.

Concerning the kinetic parameters, the glycan-free *E*. *coli* expressed KLK8 can be compared with an identical, refolded KLK8 construct, which cleaved Boc-VPR-AMC with a catalytic efficiency of about 23 300 M^−1^ s^−1^, which is nearly identical to our result (Table 1)^22^. Similarly, KLK8 from *Sf9* insect cells and yeast expression (*Pichia pastoris*) had a catalytic efficiency of 21 000 M^−1^ s^−1^ for Boc-VPR-AMC and 20 000 M^−1^ s^−1^ for the substrate H-D-IPR-pNA, respectively^7,50^. Since the KLK8 variants from eukaryotic expression are most likely glycosylated at Asn95 in the 99-loop, it seems that this modification does not influence the basic enzymatic activity, in contrast to the corresponding glycan of the KLK2 99-loop^51^. Nevertheless, the glycan carrying 99-loop, as it was observed in the mouse Klk8 structure (PDB code 1NPM) may contribute to resistance against proteolysis, protection of Cys93 against oxidation, or conformational stability. In this latter context, it may favor a distinct conformation as proposed by the conformational selection model for KLKs. In particular, the glycan at Asn95 might regulate the concerted conformational rearrangements of loops surrounding the active site and concomitantly enhance the turnover of larger polypeptides^51,52^.

**Figure 7:**
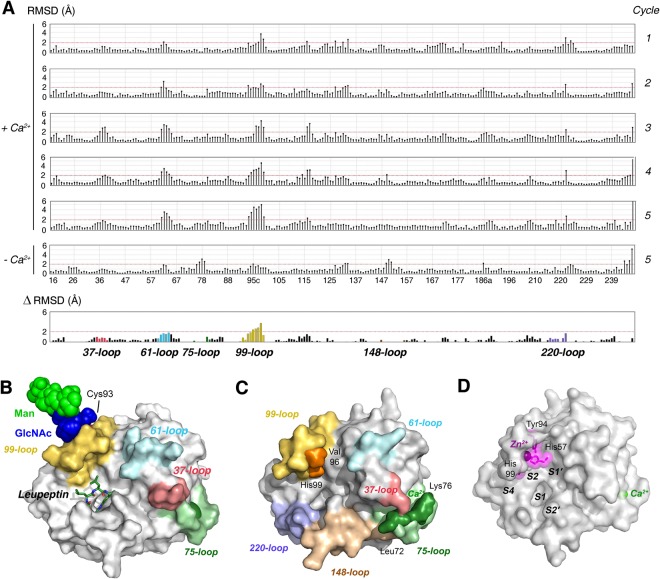
Molecular dynamics and the allosteric loop network of KLK8. (**A**) Plots of root mean square deviations (RMSD) in Å of KLK8 residue coordinates for five sequential MD production cycles at 298 K after the warm-up cycles, with respect to the final warm-up model. Data are depicted Ca^2+^ bound to the 75-loop and the last cycle without Ca^2+^ (chymotrypsinogen numbering). The significance level 2 Å (red line) is MOE default. For better visualization of loop motions, the RMSD difference (∆) of the models from cycle 5 [(+Ca^2+^) − (−Ca^2+^)] was calculated. Whereas the Ca^2+^ bound 75-loop seems more stationary, the 37-, 61-, and mainly the 99-loop exhibit large motions. This finding indicates an allosteric communication line between these loops. (**B**) Model from MD calculations of KLK8 shown as surface with colored loops with a core glycan linked to Asn95, consisting of two GlcNAc (blue spheres) and three mannoses (light green). Ca^2+^ (green) is bound to the 75-loop and leupeptin from the crystal structure is overlayed as stick model at subsites S4 to S1, in order to emphasize the wide open 99-loop (yellow) conformation, which seems to be facilitated by the presence of the glycan. The glycan may prevent cleavage in the 99-loop and disulfide formation of Cys93. (**C)** In MD calculations of glycan-free KLK8 were similar as for glycosylated KLK8. Upon removal of Ca^2+^, the 75-loop closes the substrate binding cleft in the prime side near the 37-loop. The concomitant closure of the S2 pocket by Val96 and His99 (orange) in the 99-loop might hamper binding of small substrates. The labeled loops around the active site represent the allosteric network of various trypsin-like serine proteases, which may regulate substrate access^52^. (**D**) Zn^2+^-binding would similarly close the S2 pocket and inactivate KLK8 by rotating the His57 side chain away from the catalytic triad. Such a conformation was observed in the structure of rat tonin (1TON).

In the synaptic cleft, the Ca^2+^ concentration is about 0.7 to 2 mM, which overlaps with the reported stimulatory range for KLK8 of 100 µM up to 10 mM^53^. Similar to the prototypic serine protease trypsin, the activity increases up to 4-times for small synthetic substrates by Ca^2+^ stimulation^7^. However, some coagulation factors, such as FXa exhibit more than 30-fold Ca^2+^ stimulation of their enzymatic activity against chromogenic substrates^54^. Regarding the mechanism of the stimulation molecular dynamics (MD) simulations showed that some parts of the KLK8 molecule tend to rearrange in the presence and absence of Ca^2+^ at the 75-loop (Fig. 7A). Ca^2+^ binding in the 75-loop results in significant conformational changes in the 37-, 61- and mainly the 99-loop (represented as root mean square deviations, RMSD), whereas smaller rearrangements occur in the Ca^2+^-free state. Intriguingly, MD calculations with the core glycan GlcNAc_2_Man_3_ linked to Asn95 resulted in a wide open S2 pocket, a conformation that seems to be stabilized by the glycan (Fig. 7B). This observation might explain the stronger stimulatory effect of Ca^2+^ on the activity of recombinant KLK8 from insect cells, which produce glycosylated proteins^7^. Apparently, the allosteric effect is transmitted upon Ca^2+^ binding from the 75-loop via the neighboring 37- and 61-loops to the 99-loop. A similar allostery was seen in the comparison of a Ca^2+^-bound and –free factor IXa triple mutant, with a long-ranging conformational rearrangement from the 75-loop to the center of the active site, which was termed “communication line”, involving the 148-loop, the activating N-terminal salt bridge and the S1 pocket^55^. Without Ca^2+^ the 75-loop reorients itself and covers parts of the prime side region from S6′ on, which would interfere with the binding of polypeptide substrates (Fig. 7C). Concomitantly, the 99-loop closes the upper part of the S2 subsite with Val96 and His99 in a way that would hamper substrate binding in the non-prime side, which further corroborates the importance of this alternative communication line in KLK8. Such a blocked S2 pocket, involving Lys98 and Tyr99, has been observed for factor IXa in complex with the inhibitor benzamidine in the S1 pocket, albeit with Ca^2+^ bound in the 75-loop^56^. The closed or “locked” conformation of the fIX 99-loop is based on the interaction of Tyr177 with asparagines 97 and 100, which is physiologically released by the major stimulatory factor VIIla^57^. In contrast to factor IXa, KLK8 lacks the specific residues that lock the closed conformation, as it seems to shift easier between the open and closed conformations. By contrast, mKlk8 adopts a relatively open conformation even in the absence of inhibitors or Ca^2+^. Furthermore, it has been demonstrated that Ca^2+^-binding to the 75-loop of factor IXa is allosterically linked to the Na+-binding/225-loop^58^. The corresponding 220-loop of most KLKs cannot bind Na^+^, due to the presence of cis-Pro219. A molecular dynamics study based on free and inhibitor bound KLK4 crystal structures described an allosteric interplay of loops surrounding the active site, in particular the 37-, 75-, and 220-loops, although it involves an inhibitory cation site at Glu77 and His25^59^. However, a comparative analysis of many trypsin-like serine proteases, including all known KLKs, suggested that the 99-, 148-, and 220-loop, surrounding the catalytic triad, open and close in a concerted manner, according to the conformational selection mechanism^52^. Thus, it would not be surprising, if all loops around the substrate binding cleft were connected in an allosteric network, with different characteristics in individual serine proteases.

In line with this general model, Zn^2+^ was identified as second cationic modulator of KLK8 activity, which may fix or “lock” the closed 99-loop conformation (Fig. 7D). While its physiological role at synapses is less clear than the one of Ca^2+^, its extracellular synaptic concentration is around 1 µM, but may reach much higher concentrations during zincergic signaling in the hippocampus^60^. Moreover, Zn^2+^ is a regulator of KLK5, 7, and 14 activity in skin, besides the LEKTI or SPINK inhibitors, which do not target KLK8^23,61^. Thus, Ca^2+^ and Zn^2+^ appear to be antagonistic regulators of KLK8 activity in brain and skin, especially during wound healing that is usually accompanied by an increase of the Ca^2+^ concentration^62^. By contrast, in a mouse model of spinal cord injury, mKlk8 exhibited increased expression, which was confirmed in corresponding human cases^63^. In a multiple sclerosis mouse model upregulation of mKlk8 and mKlk6 was observed in brain encephalitis and in the spinal cord during disease development to axon degeneration. Both conditions are serious medical problems, which await proper treatment, perhaps by employing KLK8 as target for pharmaceuticals^64^.

## Materials and Methods

### Expression and purification of KLK8

KLK8 cDNA was obtained from ovarian tumor tissue mRNA and cloned into the pQE30 vector at BamH/HindIII restriction sites (Qiagen). The KLK8 constructs are coding for the mature protease domain with an N-terminal Met-Arg-Gly-Ser-His_6_-tag-Gly-Ser sequence followed by an enterokinase (EK) cleavage site (Asp_4_-Lys↓Ile-Ile). Site-directed PCR mutagenesis was performed with Pfu Turbo (Stratagene) and template digestion with DpnI (New England Biolabs).

Wild-type KLK8 and the variants D70K, Y94F, H99A, and C93S were obtained from *E*. *coli* M15[pREP4] cells (Qiagen) as inclusion bodies. After treatment with denaturing lysis buffer (6 M guanidinium-HCl, 100 mM NaH_2_PO_4_, 10 mM Tris-HCl, pH 8.0) and removal of cell debris, KLK8 was purified with nickel-nitrilotriacetic acid Sepharose chromatography (Qiagen). Column washing was done by stepwise reduction of the pH from 8.0 to 5.0, followed by KLK8 elution with 8 M urea, 100 mM NaH_2_PO_4_, 10 mM Tris-HCl, pH 4.0. The solution was titrated with NaOH to pH 8.0 and incubated with 10 mM DTT overnight at 25 °C and dialyzed against the 100-fold volume of 4 M urea, 50 mM Tris-HCl, pH 8.0, 100 mM NaCl, and 0.005% Tween-20 for 12 h at 4 °C. Refolding of KLK8 was done by dropwise dilution in 2 M urea, 50 mM Tris-HCl, pH 8.0, 100 mM NaCl, 5 mM reduced glutathione, 0.5 mM oxidized glutathione, 0.002% NaN_3_, 0.005% Tween-20 in the 100-fold sample volume at 4 °C for 30 h. Afterwards, the KLK8 solution was concentrated with 10 kDa cutoff double cassette membranes. The latter procedure was repeated with 1 M urea. Final concentration in 150 mM NaCl, 50 mM Tris-HCl, pH 8.0, 0,005% Tween-20 (storage buffer) was done with microconcentrators (Vivaspin, 10 kDa cutoff, Sartorius). The N-terminal His_6_-tag was cleaved from KLK8 with EK (Sigma), of which 1 U yielded 25 μg/ml of KLK8 to 98% in 12 h at 4 °C in storage buffer, followed by treatment with the EK antibody capture kit (Sigma) for 60 min. Active KLK8 was purified with benzamidine-Sepharose affinity chromatography (Amersham Pharmacia), washed with storage buffer, containing 300 mM NaCl, and eluted with this buffer containing 20 mM p-amino-benzamidine. Eventually, size exclusion chromatography on Superdex 2000 (16/26) was performed with storage buffer. The identity of mature KLK8 was confirmed by SDS-PAGE, mass spectrometry and N-terminal sequencing.

### Positional scanning with a synthetic combinatorial peptide library

The positional scanning procedure has been previously described in detail^52^. Briefly, the diverse library with the general composition acetyl-P4-P3-P2-P1-7-ACC consists of 20 P1-, 20 P2-, 20 P3-, and 20 P4-libraries with one of the 19 canonical amino acids and norleucine (Nle) as substitute for Cys in the P1, P2, P3, or P4 positions. The three other positions contain an equimolar mixture of all these amino acids, resulting in 8000 compounds. The final concentration of activated KLK8 was 60 nM, whereby the single and total concentrations of substrates in 100 µl were 31.25 nM and 250 µM, respectively. Assays were performed in triplicate samples for 10 min at 25 °C in 150 mM NaCl, 50 mM Tris-HCl, pH 8.0, 0.005% Tween 20, and 1% dimethyl sulfoxide (DMSO), using 96-well Microfluor-1 Black U bottom plates (Dynex Technologies, Chantilly, VA) and a SpectraMax Gemini fluorescence spectrometer (Molecular Devices) with excitation at 380 nm and emission at 460 nm.

### Proteomic identification of cleavage sites

The general procedure has been described previously for KLK2, while a more detailed description on enhanced PICS was recently published^26,51^. The peptide library was generated by proteolysis of *E*. *coli* proteins by GluC. KLK8 samples were incubated with the library with a 1:300 ratio in 50 mM Tris (pH 7.5), 100 mM NaCl at 37 °C for 3 h. After the reaction, isotope labeling of protease-treated and control samples was performed, followed by liquid chromatography-tandem mass spectrometry (Q-Exactive plus MS with an Easy nanoLC 1000, Thermo Scientific). The spectrum to sequence assignment was done with X! Tandem (Version 2013.09.01) with *E. coli* strain K12 as reference proteome. Semi-specific peptides with an increase >8-fold in the KLK8 samples were accepted as cleavage products. Prime side and non-prime side sequences were assigned according to the reference database and the corresponding protease specificity presented as heat-maps employing Web-PICS^65^.

### Enzyme kinetic measurements

Prior to measurements with fluorogenic substrates, active site titration of mature KLK8 was done with pNPGB (BACHEM). The final enzyme concentration was 120 nM and the one of p-NPGB was 100 µM 150 mM NacCl, 50 mM Tris-HCl, pH, pH 8.0, 0.005% Tween-20, 1% DMSO at 25 °C. The release of p-nitrophenol was monitored at 410 nm, which allowed to calculate the molarity of active KLK8^66^. Enzymatic activity of KLK8 using AMC substrates (BACHEM) was measured in 150 mM NaCl, 50 mM Tris-HCl, pH, pH 7.5, 0.005% Tween-20, 1% DMSO at 25 °C on a Perkin-Elmer LS50B spectrofluorimeter at excitation and emission wavelength of 380 nm and 460 nm. According to the results of the specificity profiling an ideal substrate was synthesized with the formula Ac-Thr-Lys-Leu-Arg-ACC^27^. Substrate final concentrations were 40 µM and the KLK8 concentration employed was 60 nM, Ca^2+^ and Zn^2+^ were added in the range of 1 to 1000 µM and 0.5 to 500 µM, respectively.

In measurements with pNA substrates, the following KLK8 final concentrations were employed: 175 nM wt, 250 nM D70K, and 320 nM H99A in the assay buffer with 50 mM Tris-HCl pH 7.5, 100 mM NaCl, 0.005% (v/v) Tween-20, 0.02% NaN_3_, and 5% (v/v) DMSO. For determination of k_cat_ and K_M_ the concentration of Bz-Pro-Phe-Arg-pNA (BACHEM) ranged from 50 to 1000 µM, while 250 µM were used in the Zn^2+^ inhibition and Ca^2+^ activation experiments at 37 °C. The release of pNA was monitored by the absorption at 405 nm every 20 seconds for 5 minutes. The fraction of active KLK8 was determined by active site titration with BPTI (82% active) that had been titrated with trypsin, which was 48% active according to the p-NPGB burst titration^67^.

### Crystallization, data collection and processing

Using the sitting-drop vapor diffusion method, KLK8 crystals were grown at 18 °C from drops containing 1 μl protein solution (8 mg/ml), 10 mM leupeptin and 1 μl precipitant (100 mM tri-Na citrate, pH 5.6, 35% (v/v) tert-Bu-OH), equilibrated against 500 μl of precipitant solution. KLK8-Ca crystals grew in the orthorhombic space group P2_1_2_1_2_1_ with one molecule per asymmetric unit (asu), whereas KLK8-leup crystals were obtained by 2 min soaking with 100 µM ZnCl_2_ and belonged to the monoclinic space group P2_1_ with four molecules per asu, respectively (Table 2). Data for KLK8-Ca were collected with a wavelength of 0.97469 Å at the EMBL beamline X12 (DESY, Hamburg) and for KLK8-leup with a wavelength of 1.00000 Å at the beamline PX II (SLS, Switzerland). Data were indexed and integrated in XDS and scaled with SCALA and AIMLESS^68,69^. Two KLK8-Ca data sets were merged in order compensate the strong anisotropy of the 2.0 Å resolution data set. A data cutoff at 2.3 Å was suggested by the CC(1/2) values from AIMLESS. Analysis of KLK8-leup data in CTRUNCATE and XTRIAGE showed a pseudomerohedral twin fraction of 0.24, which was taken into account in following steps of refinement^70,71^.

**Table 2:** Data collection and refinement statistics.

	KLK8-Ca	KLK8-leup
**Data collection**		
Space group	*P2*_*1*_ *2*_*1*_ *2*_*1*_	*P2*_1_
**Cell dimensions**		
*a*, *b*. *c* (Å)	46.00, 51.74, 82.86	83.10, 46.04,103.35
*α*, *β*, *γ* (°)	90.00, 90.00, 90.00	90,00, 91.40, 90.00
Resolution (Å)	50.0 − 2.30 (2.38 − 2.30)*	83.10 − 2.10 (2.21 − 2.10)*
R_merge_/R_pim_ (%)	15.3 (37.2)/7.0 (23.0)	17.8 (38.0)/10.6 (26.7)
I/σ	8.1 (3.3)	6.1 (2.8)
Completeness (%)	99.7 (97.3)	97.7 (93.2)
Average multiplicity	9.3 (6.6)	3.4 (2.6)
CC(1/2)	0.995 (0.966)	0.975 (0.705)
**Refínement**		
Resolution in refinement (Å)	43.9 − 2.30 (2.38 − 2.30)	65.53 − 2.10 (2.15 − 2.10)
Completeness (%)	99.6 (98.6)	97.7 (88.3)
No. reflections/reflections test set	9190 (872)/435 (44)	45152 (2508)/2354 (122)
R_work_/R_free_ (%)	22.1 (30.0)/26.6 (33.2)	21.2 (26.5)/25.9 (27.0)
**No. atoms**		
Protein	1711	6844
Ligand/ion	1 (Ca^2+^)	120 (Leup)/4 (Ca^2+^)
Water	84	1137
***B*** **-factors, (Å** ^**2**^ **)**		
Protein	29.4	15.0
Ligand/ion	26.2 (Ca^2+^)	20.1 (Leup)/29.5 (Ca^2+^)
Water	29.0	21.9
**R. m. s. deviations**		
Bond lengths (Å)	0.006	0.004
Bond angles (°)	0.721	0.771
**PDB accession code**	5MS3	5MS4

^*^Highest resolution shell in parentheses.

### Molecular replacement, model building, and refinement

For the KLK8-Ca data in space group P2_1_2_1_2_1_ a molecular replacement search was performed with PHASER in the automated search mode using the mouse Klk8 model with 73% identical residues (PDB code 1NPM)^72^. The best solution with one mol/asu exhibited Z values for the rotation function (RFZ) of 17.0 and of 29.3 for the translation function (TFZ) with a log-likelihood gain (LLG) of +970 and an R-factor after rigid body refinement of 52.2%. After inspection of proper molecular packing, the solution was evaluated with a total omit map from SFCHECK, which had a figure-of-merit (FOM) = 80.3%^73^. In case of the KLK8 data in space group P2_1_, the refined polypeptide KLK8-Ca model was employed in PHASER auto mode, resulting in a LLG = +6792 with Z-values of RFZ = 18.5 and TFZ = 21.8, and an R-factor of 44.0%.

Model building for KLK8-Ca was done iteratively with COOT and refinement with PHENIX, resulting in final R_cryst_ and R_free_ values of 22.4% and 26.6%, respectively, for a maximum resolution of 2.30 Å (Table 2)^71^. The Ca^2+^ site was fully occupied, while no significant electron density was observed for leupeptin. Due to the high anisotropy, real space refinement was required to reduce RSRZ outliers to acceptable 8.8%. Asn and Gln side chain orientations were corrected according to NQ-Flipper V2.7^74^. Similarly, the model of KLK8-leup was refined, requiring the pseudomerohedral twin operator (h, -k, -l), resulting in R_cryst_ and R_free_ values of 21.2% and 25.9%, respectively, at a resolution of 2.1 Å (Table 2). An anomalous Fourier map showed no distinct peaks corresponding to potential Zn^2+^ sites, as well as density correlation and real space R-factors from OVERLAPMAP in CCP4i did not confirm transition metal ions. Overall, the main chain of all polypeptides is well defined, except for the loop around Arg148 and two C-terminal residues. Also, the 75-loops of KLK8 copies C and D are not well defined, while leupeptin shows some variations in the individual Leu side chain positions and the acetyl group. The Ramachandran plots show 97.3% (KLK8-Ca) and 94.9% (KLK8-leup) of residues in the most favored regions and 2.7% and 5.1% in additionally allowed regions, while no outliers were observed. All figures were created with PyMOL v1.7rc1 and electrostatic potential phi maps were calculated with the PDB2PQR Server and APBS tool^75^ (CIT). Root mean square deviations (RMSD) were calculated with the program SUPERPOSE^76^.

### Molecular dynamics in solution and docking calculations

To perform the molecular dynamics calculations in an aqueous solution environment model coordinates were first titrated at pH 7.0 using the Protonated3D function of MOE 2015.10 (www.chemcomp.com)^77^. The resulting structure was solvated in a cubic box of water molecules with specified edges (83.6 Å × 83.6 Å × 83.6 Å) centered on the KLK8 using periodic boundary conditions. Cl^−^ counter ions were added to maintain overall neutrality. Afterwards, a series of equilibration steps were performed by molecular dynamics annealing runs, without using a barostat, for 100 ps at temperatures 50 K, 150 K, 200 K, 250 K and 298,15 K. The production was performed for 500 ps at 298.15 K. The molecular dynamics calculations were accomplished using the AMBER99 force field as implemented in NWChem 6.6^78^. During warm up 10000 replicas were collected at each temperature and 5 × 100000 replicas during each production cycle. In each case the time step was 0.001 ps. After each warm-up cycle and each production cycle a PDB-structure was generated and used for further analysis. The single replicas were not compared with each other. Unbound molecular docking to KLK8 was similarly performed, using a model based on the PAR-1 fragment in complex with thrombin (PDB code 1LU9) and the PICS results. The coordinates of the best hit with acceptable binding of P1-Arg to the S1 pocket were further optimized with MOE.

### Accession codes

Mass spectrometry proteomics data for PICS were deposited to the ProteomeXchange Consortium via the PRIDE archive with the dataset identifier PXD006884. Coordinates have been deposited at the Protein Data Bank (www.rcsb.org) under the accession numbers 5MS3 (KLK8-Ca) and 5MS4 (KLK8-leup).
